# Rapid Amygdala Responses during Trace Fear Conditioning without Awareness

**DOI:** 10.1371/journal.pone.0096803

**Published:** 2014-05-13

**Authors:** Nicholas L. Balderston, Douglas H. Schultz, Sylvain Baillet, Fred J. Helmstetter

**Affiliations:** 1 Department of Psychology, University of Wisconsin-Milwaukee, Milwaukee, Wisconsin, United States of America; 2 Department of Neurology, Medical College of Wisconsin, Milwaukee, Wisconsin, United States of America; 3 McConnell Brain Imaging Centre, Montreal Neurological Institute, McGill University, Montreal, Qc, Canada; University College of London - Institute of Neurology, United Kingdom

## Abstract

The role of consciousness in learning has been debated for nearly 50 years. Recent studies suggest that conscious awareness is needed to bridge the gap when learning about two events that are separated in time, as is true for trace fear conditioning. This has been repeatedly shown and seems to apply to other forms of classical conditioning as well. In contrast to these findings, we show that individuals can learn to associate a face with the later occurrence of a shock, even if they are unable to perceive the face. We used a novel application of magnetoencephalography (MEG) to non-invasively record neural activity from the amygdala, which is known to be important for fear learning. We demonstrate rapid (∼170–200 ms) amygdala responses during the stimulus free period between the face and the shock. These results suggest that unperceived faces can serve as signals for impending threat, and that rapid, automatic activation of the amygdala contributes to this process. In addition, we describe a methodology that can be applied in the future to study neural activity with MEG in other subcortical structures.

## Introduction

During Pavlovian fear conditioning an initially innocuous stimulus (conditional stimulus; CS) is repeatedly paired with an aversive outcome (unconditional stimulus; UCS) [1]. This relatively simple learning event engages multiple psychological and physiological response systems. For example, individuals quickly acquire the ability to explicitly state the nature of the cue-outcome relationship during training. At the same time they develop a conditioned emotional response (CR) that can be expressed later when they again encounter the danger signal [2], [3].

When the CS and the UCS are separated by a temporal gap, as in the “trace” conditioning procedure, people can only develop conditioned responses if they are also able to explicitly state, and are thus consciously aware of, the contingent relationship between stimuli [4]–[6]. Based on these results it is assumed that because subjects are not directly experiencing the CS during the presentation of the UCS, they must be able to accurately maintain the CS in memory until the presentation of the UCS in order to bridge the temporal gap between these two stimuli [4], [5].

In this paper we challenge the generality of this assumption by training subjects to fear unperceived faces. There is a broad literature suggesting that the amygdala shows a specific sensitivity to faces and other “prepared” stimuli [7]–[12], that these stimuli are better at predicting aversive outcomes than other signals [13]–[16], and that training with these stimuli lead to a fear memory that is more difficult to extinguish [16], [17].

Although fearful or angry stimuli are often thought of as “prepared stimuli,” recent work from our lab suggests that even neutral faces strongly activate the amygdala when novel [18], suggesting that one function of the amygdala may be to evaluate faces for evidence of threat. In this experiment we tested they hypothesis that, this intrinsic face processing, which has been shown to operate independent of awareness [11], [19]–[25], may be sufficient to support trace conditioning without awareness (For a counter argument see [26], [27]). Here we show that unperceived faces evoke rapid amygdala responses that persist even after the face is no longer present, and that these responses may be sufficient to bridge the temporal gap between CS and UCS during trace fear conditioning.

## Methods and Materials

### 2.1 Participants

Nineteen (10 female) neurologically healthy University of Wisconsin-Milwaukee students (Age: *M* = 22.17, *SD* = 3.23) participated. All subjects received extra credit in their psychology courses, $30, and a picture of their brain. Of the original 19 participants, 9 were in the unfiltered group, 9 were in the filtered group, and 1 was excluded from the analysis due to excessive head movement. Additionally, two participants were excluded from the testing phase analysis because of equipment failure.

#### 2.1.1 Ethics statement

All participants gave written informed consent prior to participation, and the protocol was approved by the Institutional Review Boards for human subject research at the University of Wisconsin-Milwaukee and the Medical College of Wisconsin.

### 2.2 Procedure

The first goal was to show that the subjects could implicitly learn trace fear conditioning with masked face CSs in the absence of awareness. The second goal was to test the hypothesis that this learning without awareness during trace fear conditioning was specific to face CSs. The final goal was to test the hypothesis that this learning without awareness during trace fear conditioning is supported by rapid activation of the amygdala.

We exposed subjects to differential trace fear conditioning with masked face CSs while we recorded their brain activity using magnetoencephalography (MEG; For a detailed description of the current methodology and video demonstration, see [28]). During training subjects received 120 presentations of face stimuli which lasted for 30 ms and were immediately followed by an 870 ms presentation of a masking stimulus. Subjects were split into 2 groups: our experimental group saw broad spectrum faces (*Unfiltered*), while our control group saw high-pass filtered faces (*Filtered*). We chose high-pass filtered faces as control stimuli because they possess many of the same physical features as broad spectrum faces, but have been previously shown not to drive BOLD responses in the amygdala [29]. Each subject saw two faces, one of which was always followed by a shock (CS+), one of which was never followed by a shock (CS−). Training occurred across 4 blocks of 30 trials (15 per CS type). Trials were separated by a 6 s intertrial interval (ITI), to minimize the effect of the shock on recordings during subsequent trials. During training, subjects continuously rated their expectancy of receiving the shock. Subjects were also asked to rate the intensity of the shock after each of the training blocks. Trial order was counterbalanced across subjects. We also measured heart rate (ECG), eye movements (EOG), skin conductance level (SCL), and head position relative to fiducial points (nasion, left and right tragi, and 50–100 scalp points). After the conditioning session, subjects were escorted to the MRI scanner at the Medical College of Wisconsin, where we began by collecting high resolution MRI images to model the sources of the MEG signal.

Because the SCR resolves of a timescale much longer than the CS-UCS interval used in this experiment [30], we were not able to directly assess implicit learning during the conditioning phase. Thus, we used a subsequent reacquisition phase to indirectly determine whether subjects were able to learn the contingencies during the training phase. This reacquisition test took place following the anatomical MRI scans, while the subject was still in the MRI scanner. Although fMRI data were collected, these data will not be reported here. During the reacquisition test subjects were exposed to 6 (24 trials total, 6 per trial type), 8 s presentations of new and old pictures. Old pictures kept the same picture-shock associations. For comparison, one new picture was paired with a shock, while the other was presented without the shock. In addition, subjects assigned to the Unfiltered group saw unfiltered new and old faces, while subjects assigned to the Filtered group saw filtered new and old faces. Trials were separated by a 20 s intertrial interval, to allow enough time for the SCR to return to baseline. Trial order was counterbalanced across subjects.

If subjects learned the face-shock pairings, they should show two patterns of behavior during the reacquisition session. First they should show differential responses to the training phase stimuli on the first re-exposure trial. Second, they should show larger differential responses to the old stimuli than to the new stimuli during the subsequent reacquisition trials. We tested these possibilities independently.

### 2.3 Stimuli

The neutral expressions from 4 models (2 female) were chosen from the Pictures of Facial Affect (POFA) database [31]. Broad spectrum images remained unfiltered. High spatial frequency images were passed through a 5 cycle/degree high pass filter. Neutral expressions from six separate models were merged to create the mask, which was presented to all participants on all trials. The CSs and mask were then aligned so that the eye region of the face was in the same position across images, cropped using an oval template so that only the face was visible, and normalized so that each image had the same mean luminance.

### 2.4 Electrical Stimulation

Constant AC (60 Hz) electrical stimulation was administered from an internally isolated source (Contact Precision Instruments, Model SHK1, Boston, MA), via surface cup electrodes (Ag-AgCl, 8 mm diameter, Biopac model EL258-RT, Goleta, CA) filled with electrolyte gel (Signa Gel, Parker laboratories Fairfield, NJ). We placed the electrodes over the subject’s right tibial nerve, and calibrated the stimulation prior to the experiment so that the subject rated the intensity as painful but tolerable.

### 2.5 Skin Conductance Responses

SCRs were recorded at 200 hz throughout testing via two surface cup electrodes (Ag-AgCl, 8 mm diameter, Biopac model EL258-RT, Goleta, CA) filled with electrolyte gel (Signa Gel, Parker laboratories Fairfield, NJ) attached to the bottom of the participants left foot, approximately 2 cm apart. We sampled SCL during the CS period, and the preceding two second baseline. Values were converted to a percent change from the baseline, and the maximum value during the CS period was used to indicate the response magnitude.

### 2.6 UCS Expectancy

Throughout training and testing, subjects continuously rated their expectancy of receiving the electrical stimulation, using a rating scale at the bottom of the screen. Subjects placed the cursor near 0 if they were absolutely sure that they would not receive a stimulation, 100 if they were absolutely sure they would receive the stimulation, and 50 if they were unsure whether or not they would receive the stimulation. Responses were recorded throughout the experiment and sampled at 40 Hz. During the training phase, we sampled the responses for 1 second after the onset of the CS. During the testing phase, we sampled the responses during the last 4 seconds of the CS period.

### 2.7 MRI

We conducted whole brain imaging using a 3T long bore GE Signa Excite MRI system. We acquired high resolution spoiled gradient spoiled gradient recalled (SPGR) acquisition images to serve as anatomical maps for the MEG source imaging analysis. We imported the SPGR volume into Brainstorm [32] where we transformed it into Talairach space and identified the fiducial points. We used the Freesurfer software package [33]–[35] and 3dSlicer [36] to create the surface models of the cortex, amygdala, and hippocampus. We then imported these surfaces into Brainstorm [32], where they were manually aligned with the SPGR volume, and downsampled to 15,000, 1,000 and 2,000 vertices (dipoles), respectively [28].

### 2.8 MEG

#### 2.8.1 Acquisition

We acquired the MEG data at 2 khz using the Elekta-Neuromag VectorView MEG system at Froedtert Hospital. The system has 306 sensors, grouped into triplets consisting of 2 orthogonal planar gradiometers and 1 planar magnetometer, and measures magnetic flux at 102 positions. Recording took place in light magnetically-shielded room with the Elekta-Neuromag MaxField active shielding system. We verified head position at the start of each of the four runs using the HPI coils.

#### 2.8.2 Preprocessing

Raw data were initially processed using Elekta-Neuromag’s MaxField software, which uses signal source separation to attenuate signals from far-field sources [37], [38]. We used MEG clinic [39] to identify heartbeats and eyeblinks, and visually inspected the results. Next we performed a principle components analysis, and created signal space projection (SSP) vectors corresponding to the artifact correlated with each type of event. These SSP vectors were then factored out of the MEG recordings, which were then band-pass filtered (1–200 Hz). Next we extracted the single trial data, which consisted of a 200 ms pre-CS baseline period and 900 ms CS/mask period, and averaged across trials based on CS type [28].

We imported both trial averages and the single trial recordings into Brainstorm [32]. First we aligned the recordings with the SPGR volume using the fiducial points. Then we refined this alignment by using the points collected along the individual’s scalp. Next we computed the forward model using an overlapping spheres approach, taking the cortical surface as its input [40]. Afterward we computed the noise covariance matrix, using the baseline period for each trial as input [41]. Finally, we computed the inverse model using the minimum norm estimate approach [42], and estimated the amplitude for each of the 18,000 current dipoles distributed across the cortical (cortex, amygdala, and hippocampus) surface [28]. After this initial preprocessing, we conducted two parallel analyses to investigate evoked responses and induced oscillations.

#### 2.8.3 Evoked responses

The source maps for the *trial averages* were normalized and used in source level group analyses. Trial averages were first band-pass filtered (1–20 Hz), and converted to absolute values. Next, they were converted to Z-scores based on the mean and variability in the baseline period. The resulting maps were spatially smoothed using a (σ = 5 vertices), and projected onto the default cortical surface for the study. These normalized source maps were then used for group-level t-tests, which are described in the results section.

Given that statistical tests performed at the source level require a large number of multiple comparisons, uncorrected p-values are not appropriate. Therefore, we used *Monte Carlo* simulations to correct for multiple comparisons, similar to AFNIs 3dClustSim program [43]. This approach is based on the assumption that valid statistical results will tend to be spatially contiguous, while Type I errors will be randomly distributed across the sample space. For each simulation we generated a set of random p-values across the vertices of the cortex used in our group-level analysis. We then spatially smoothed this random p-value map using the same Gaussian kernel used on the group-level data. Next we applied our alpha threshold to the map, and identified the largest cluster of spatially contiguous vertices above this threshold. We then recorded this value as the maximum cluster size for this simulation. After repeating the simulation 10,000 times, we were able to create a probability distribution of maximum cluster sizes, given our alpha threshold. From this distribution we were able to estimate how likely it would be for a cluster of a given size to emerge due to chance alone. Of the 10,000 simulations, we found a maximum cluster size of 7, which was reached only once. Therefore, given an alpha threshold of 0.05 and a minimum cluster size greater than 7, we estimated that our corrected p-value was less than 0.0001. In the end, we chose a more conservative minimum cluster size threshold of 10 connected vertices, and added a minimum time constraint of 20 ms as well.

#### 2.8.4 Induced oscillations

In the results section we describe the evoked analysis that we used to identify a functional region of interest (ROI) in the amygdala. We wanted to further characterize the temporal and spectral components of this signal. Toward this aim, we computed time frequency decompositions on the sources for this ROI for the individual trials. First we projected the source maps onto the default anatomy, and identified the ROI. Next we computed the time frequency decomposition by convolving the signal averaged across the vertices within the ROI with a complex morelet wavelet, with a carrier frequency of 1 Hz and a time resolution of 3 s FWHM [44]. We averaged the resulting time frequency maps across trials, and converted the values to Z-scores based on the variability in the baseline period. The resulting normalized time frequency maps were then used for group-level analyses.

Similar to the source analysis, the analysis of the time frequency maps also requires a large number of multiple comparisons. Also similar to the source analysis, we expected valid statistical results to form contiguous clusters within the time frequency maps. Therefore, we again used *Monte Carlo* simulations to correct for multiple comparisons. However, because the smoothness of the experimental data is inherently dependent upon the frequency of the signal, it is difficult to simulate a random set of p-values that would match the smoothness of a typical time frequency map. Therefore, for each simulation we chose to shuffle the p-values that we actually observed in our statistical comparison in the time, but not the frequency domain. We then identified the largest cluster of contiguous p-values within the simulated map that surpassed our alpha threshold of 0.05. We repeated this process 10,000 times and built a distribution of these maximum cluster sizes. We found that cluster sizes >14 contiguous supra-threshold p-values occurred on less than 0.1% of the simulations. Therefore, given an alpha threshold of 0.05 and a minimum cluster size >14 contiguous p-values, we estimated that our corrected p-value was <0.001.

## Results

### 3.1 Learning without Awareness

The first goal was to show that trace conditioning with masked face CSs was possible even without awareness. Therefore, the first thing we needed to show was that subjects in this experiment were unable to become aware of the experimental contingencies. During the training session, the pattern of shock expectancy did not differ between the CS+ and the CS− (*F*(1,34) = 0.104, *p* = 0.75), suggesting that subjects were unaware of the experimental contingencies.

**Figure 1:**
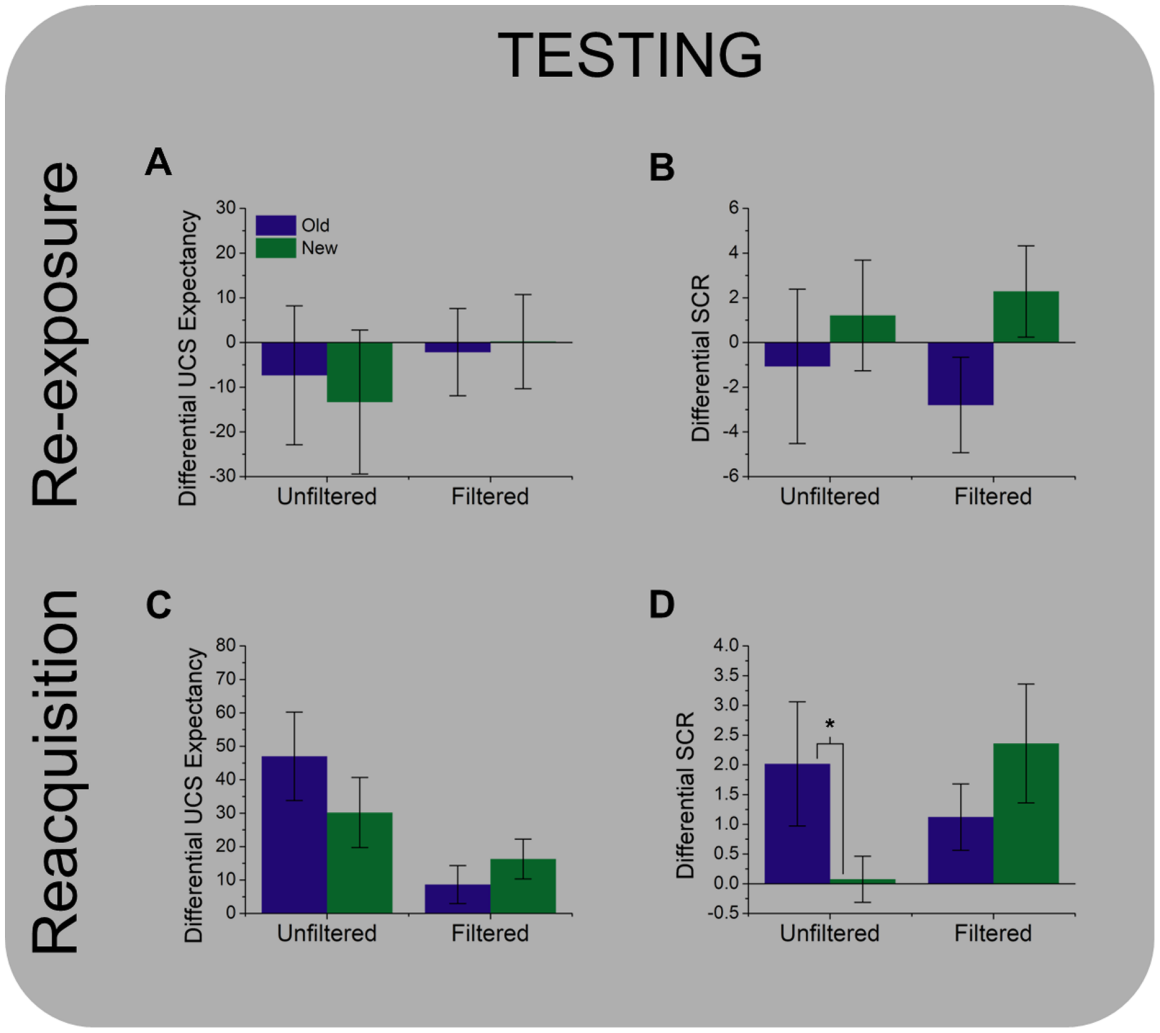
Trace fear conditioning with masked face CSs affects performance in a subsequent reacquisition task. During the training session, subjects were presented with masked filtered and unfiltered face stimuli. Stimuli were presented for 30+ trials. (**A, B**) Trace fear conditioning with masked face CSs does not appear to affect differential UCS expectancy (**A**) or differential SCR expression (**B**) during a subsequent unmasked re-exposure to the stimuli. **C.** Similarly, trace fear conditioning with masked face CSs does not affect subsequent differential UCS expectancy during a subsequent reacquisition session. **D.** In contrast, trace fear conditioning with masked unfiltered, but not filtered faces, affects subsequent differential SCR expression during a subsequent reacquisition session. Bars represent the mean±SEM. (*p<0.05).

Next we needed to determine whether or not the training impacted subjects’ performance during the subsequent reacquisition session. First we looked at the shock expectancy and SCR data from the re-exposure trial. We performed a 2 (New vs. Old)×2 (CS+ vs. CS−) ANOVA on these values for the Unfiltered and Filtered groups. Surprisingly we found no evidence of differential responding for the Old stimuli on either measure for either group (*ps* >0.05; Figure 1a–b); however, we noticed that our SCR values were subject to a high degree of variability. Given that we used a within subject design with a relatively large number of stimulus types, we suspected that the initial SCR values may have been influenced by the order in which these stimuli were presented. Although the trial sequence was counterbalanced, we had a relatively small number of subjects relative to the number of possible trial sequences. Therefore, to determine whether SCR magnitude was influenced by trial sequence, we correlated the magnitude of the SCR with the order of each trial within the sequence, and found that these were in fact negatively correlated (*r*(64) = −0.34, *p* = 0.007). This within phase habituation is commonly seen in SCR studies, and tends to be most prominent on early trials [45].

After looking at the data for the initial re-exposure trials, we looked at the responses from the reacquisition trials, those after the first CS-UCS pairing. As with the re-exposure trial, we analyzed shock expectancy and SCRs, and performed a 2 (New vs. Old)×2 (CS+ vs. CS−) ANOVA on these values for the Unfiltered and Filtered groups. Unlike the re-exposure trial we found a significant Group×CS interaction for the SCRs in the Unfiltered group (*F*(1,7) = 5.941, *p* = 0.045; Figure 1d). Post hoc stats suggest that the Unfiltered group showed a significantly larger differential SCR to the Old stimuli than to the New (*t*(7) = 2.25, *p* = 0.045), suggesting that the training phase CS-UCS pairings affected their testing phase performance. This interaction was not present for the Filtered group (*p*>0.05). Additionally, neither group showed a significant interaction for the shock expectancy measure (*ps* >0.05; Figure 1c), although both groups showed evidence of differential shock expectancy (Unfiltered: *F*(1,7) = 9.67, *p* = 0.017; Filtered: *F*(1,7) = 6.36, *p* = 0.045). These results suggest that the CS-UCS pairing affected testing phase implicit performance only for the group shown the unfiltered faces, and that this effect was not accompanied by a comparable change in explicit performance.

As with previous studies our results suggest that subjects can learn to associate a stimulus with a shock that overlaps in time, even though they are unable to consciously identify the stimulus [4], [30], [46]. However, contrary to previously published work [4]–[6], we show that individuals can also associate a stimulus with a non-contiguous shock, even though they are unable to consciously identify the stimulus. We believe that this discrepancy arises from the fact that we used faces as our conditional stimuli. Threats in the environment evoke fearful facial expressions, and these expressions can alert conspecifics to the presence of the threatening stimulus [47]. Therefore, an efficient threat detection module should be capable of rapidly identifying a face, an automatically processing its expression [12]. The amygdala, which is critically important for fear learning, is also particularly sensitive to faces [48]. Additionally, this structure shows differential responses to emotional facial expressions even if the individual is unable to consciously identify the face [22]. Therefore, the amygdala might be capable of contributing to a face representation that persists across a brief interval of time, potentially supporting trace conditioning without awareness. If this is the case, then we should see larger amygdala responses to face stimuli than to non-face stimuli during trace conditioning without awareness.

### 3.2 Faces Rapidly Activate the Amygdala

The third goal was to test the hypothesis that trace fear conditioning with masked face CSs is mediated by rapid activation of the amygdala. We performed a series of t-tests on the normalized source maps generated from the trial averages described in Section 2.5.3: First we compared the respective overall responses to the unfiltered and filtered faces. Next we compared the overall responses to the CS+ and CS−. Finally, we compared the difference between the CS+ and CS− across groups.

**Figure 2:**
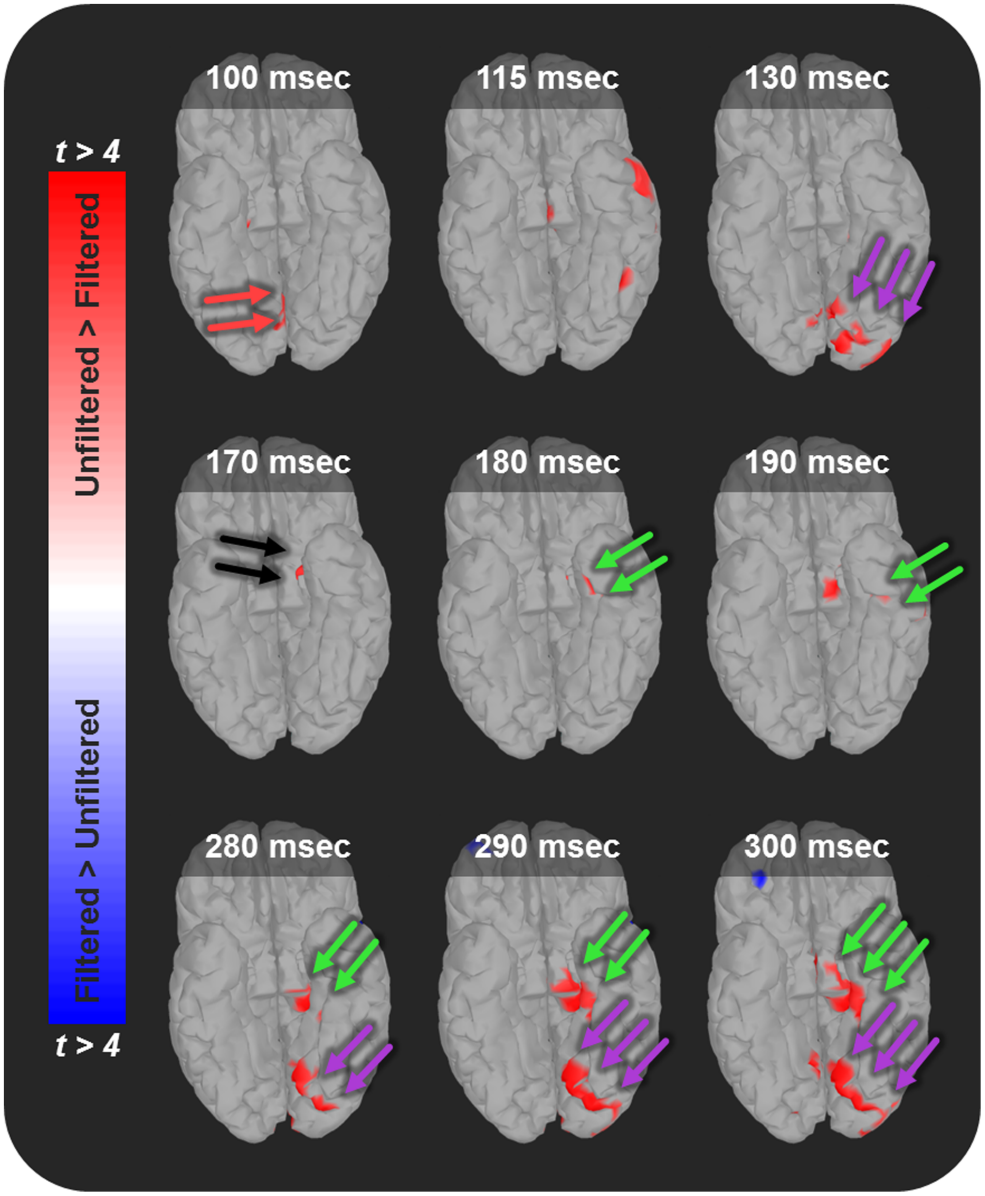
Unfiltered faces evoke larger responses than filtered faces in a network of brain regions important for face perception. Early responses to unfiltered faces seem to be predominantly in occipital regions. Amygdala activity emerges at approximately 170>Filtered t-test at the corresponding dipole. Warm colors represent larger responses to unfiltered faces than to filtered faces. Cool colors represent larger responses to filtered faces than to unfiltered faces. Arrows highlight regions of interest discussed in text (red = V1; purple = occipital face area/lingual gyrus; black = amygdala; green = medial temporal lobe).

A number of regions showed differential responses to unfiltered and filtered faces. These include the primary visual cortex, the occipital face area, the hippocampus/parahippocampal gyrus, the occipitotemporal gyrus, and the amygdala (See Figure 2 and Video S1). In all but the occipitotemporal gyrus, responses to unfiltered faces evoked larger responses than filtered faces.

**Figure 3:**
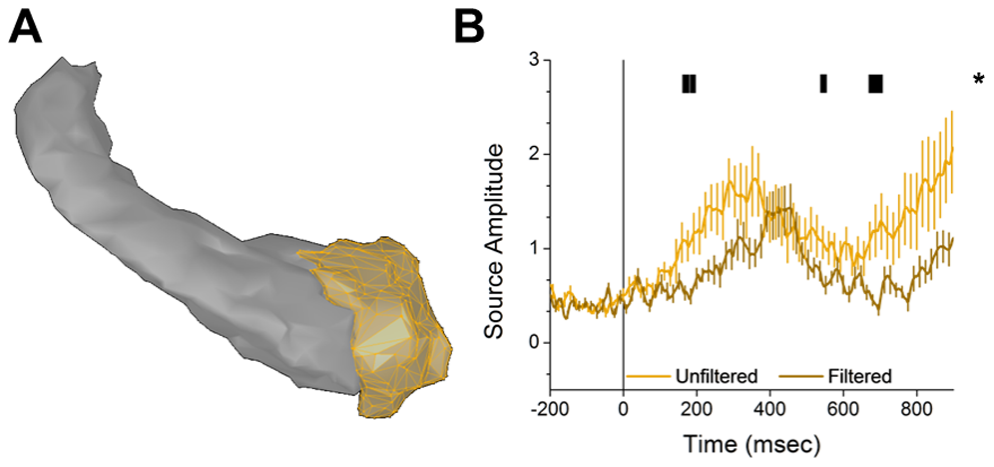
Unfiltered faces evoke significantly larger amygdala responses than filtered faces. **A.** 3d surface model of the amygdala and hippocampus of a representative subject (amygdala vertices shown in orange). **B.** Timecourse of amygdala responses evoked by the unfiltered (light orange) and filtered (dark orange) faces. Lines represent the mean±SEM. (* = *p*<0.05).

Consistent with our hypothesis, we found a cluster of activation in the left amygdala that showed significantly larger responses to unfiltered faces than to filtered faces. To understand the temporal dynamics of the amygdala responses, we plotted the mean and standard error of the mean in Figure 3b. The amygdala response to unfiltered faces had an early onset (as early as 165 ms), and grew significantly larger than the response to filtered faces. In addition, the response to unfiltered faces appeared to be bi-phasic and larger than the response to filtered faces, both early and late in the trace interval (Figure 3b).

### 3.3 Human Faces Evoke Bursts of Gamma Activity in the Amygdala

The classical approach to analyzing stimulus-evoked responses with MEG captures signals that are consistently in-phase across trials. However, induced bursts of event-related gamma activity maybe undetected following this approach because of jittering latencies and phases across experimental repetitions [49]. Gamma oscillations are involved in feature binding in object perception [49], among other cognitive processes [50], and have been detected in the amygdala using depth electrodes [51], [52]. We therefore computed the time-frequency decomposition of the amygdala source time series to detect possible event-related power changes at specific frequencies within the [10], [90] Hz range.

**Figure 4:**
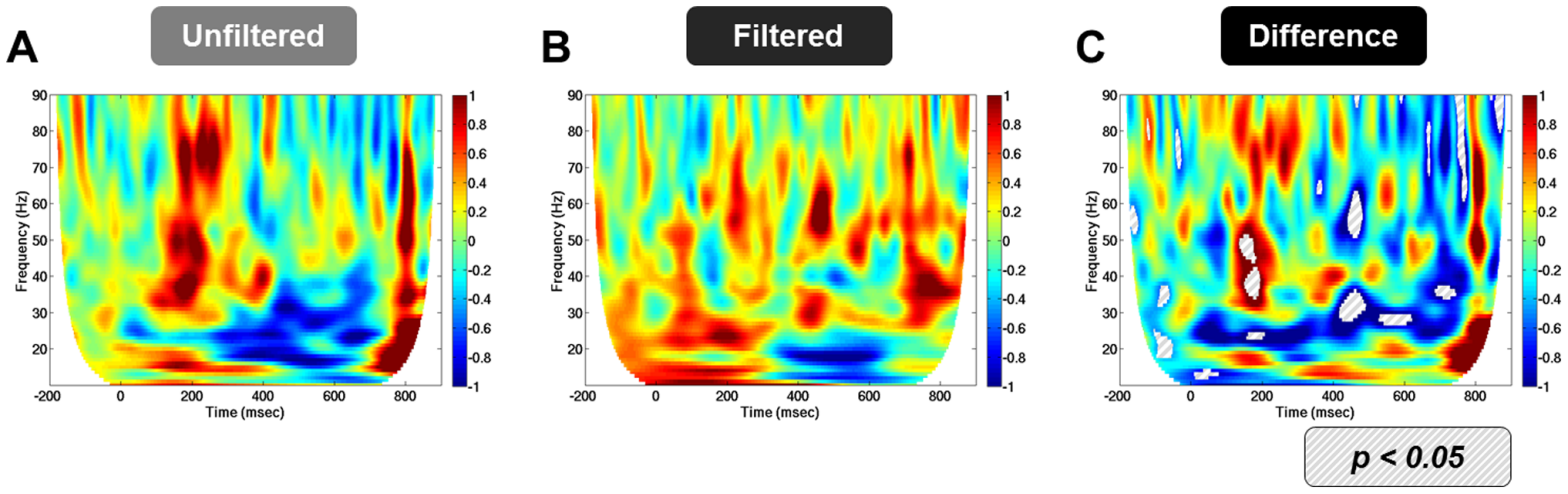
Unfiltered faces evoke bursts of gamma activity at ∼170 msec. (**A, B**) Time frequency maps evoked by unfiltered (**A**) and filtered (**B**) faces. Warm colors represent an increase in power above baseline. Cool colors represent a decrease in power below baseline. **C.** Time frequency map of difference scores for the Unfiltered>Filtered comparison. Warm colors represent Unfiltered>Filtered value. Cool colors represent Filtered>Unfiltered value. Hatched fill represents a significant difference in either direction.

We sampled the time course of activity from the amygdala region of interest for each trial, and decomposed each of these time series into their individual spectral components. Using these maps, we computed t-tests for the following comparisons: unfiltered>filtered, CS+>CS−, unfiltered (CS+ – CS−)>filtered (CS+ – CS−). As with the previous analyses, significant differences were found only with the unfiltered>filtered comparison. Interestingly, the unfiltered group showed increases in power in the gamma frequency band at a latency of approximately 170 ms, which the filtered group did not reveal (Figure 4). Consistent with the results from the evoked analysis, these results suggest that the amygdala shows a specific sensitivity to faces, even if they are presented below the threshold for conscious detection.

### 3.4 Areas Lateral to the Amygdala cannot Account for the Signal Observed in Amygdala Sources

Given that signal to noise ratios are larger for MEG signals localized to deep sources tend to be rather small [53], we wanted to be sure that the signals that we observed in the amygdala were specific to this region, and not an artifact generated by neural activity from regions closer to the MEG sensors. If the responses that we observe in the amygdala are due to poor source localization, we should see regions around the amygdala showing 1) larger evoked responses for Unfiltered faces at ∼170 ms and 2) bursts of gamma activity at ∼170 ms.

**Figure 5:**
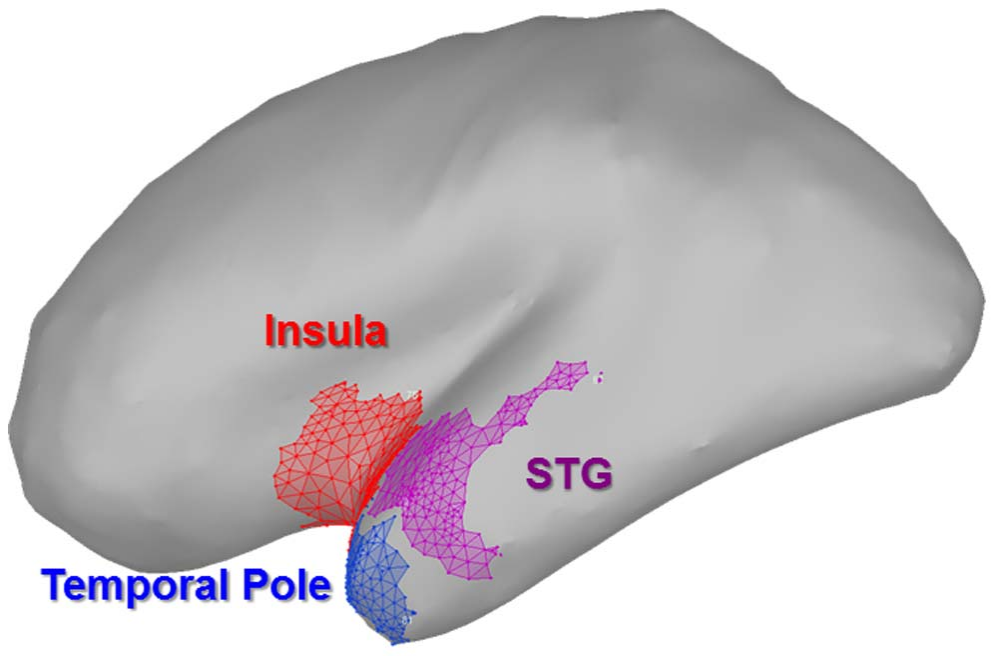
Regions identified as potentially contributing to signal observed in the amygdala. We used a rigorous and unbiased approach to identify potential sources of contamination. Of all the regions sampled, these are the only regions to potentially show evidence of gamma oscillations at ∼170 ms. Therefore they were subjected to further analysis.

We used a rigorous and unbiased approach to identify potential sources of contamination. First, we used Brainstorm’s surface clustering algorithm to generate a random set of 100 ROIs across the cortical surface. We then narrowed our analysis to the ROIs within the anterior temporal lobe (i.e. those nearest to the amygdala). We then computed the time frequency decompositions on the trial averages within these regions to identify possible evidence of gamma oscillations in sources surrounding the amygdala. We observed some evidence of gamma oscillations in 3 regions lateral to the amygdala (Figure 5), and therefore computed time frequency decompositions on the individual trial data, as described in the above section.

**Figure 6:**
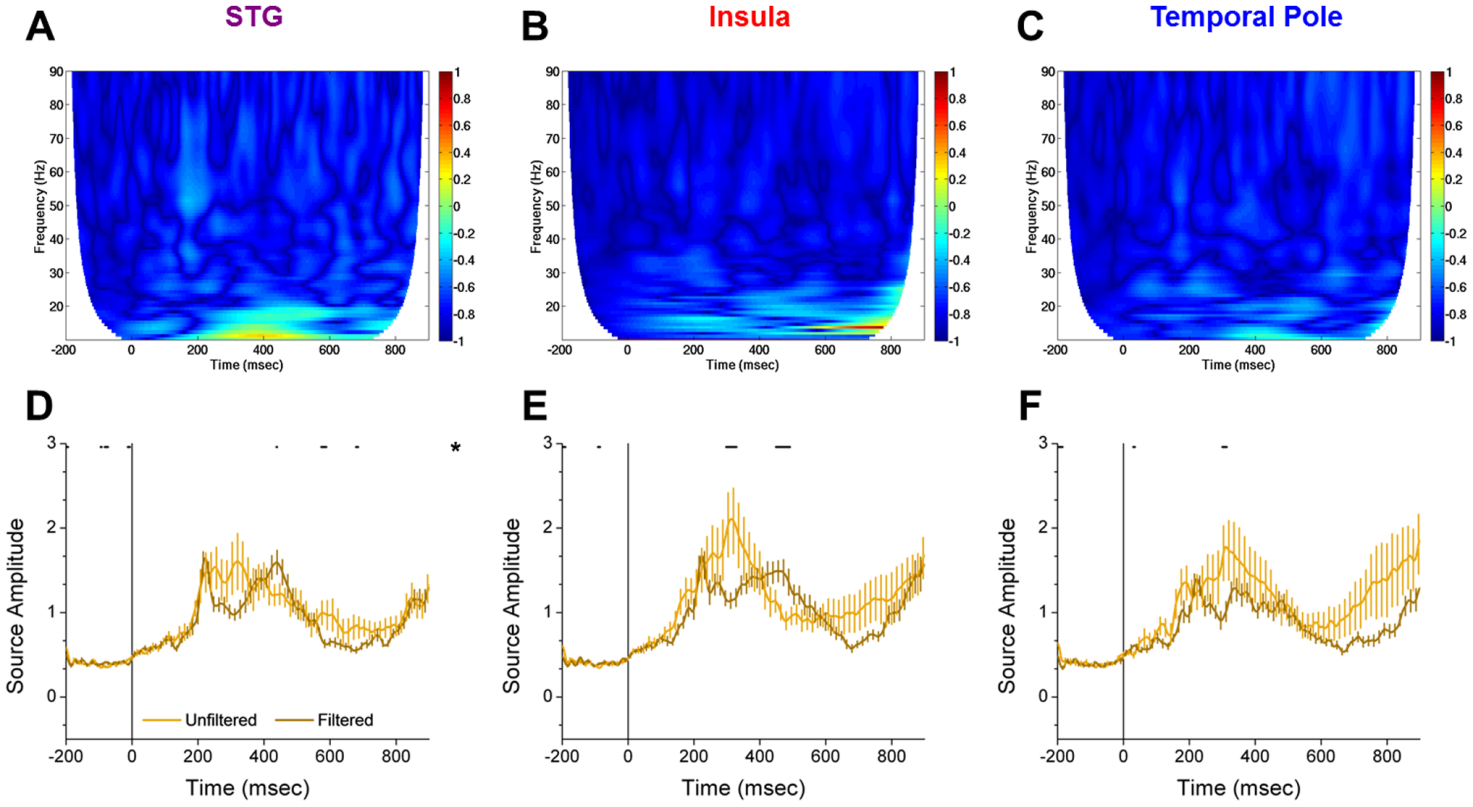
Regions identified do not contribute to signal observed in the amygdala. (**A–C**) Differential spectrograms for the regions identified in Figure 6. Unlike amygdala sources, these regions do not show evidence of differential gamma oscillations at ∼170 ms. (**D–F**) Similarly, these regions do not show larger evoked responses for unfiltered than for filtered stimuli at ∼170 ms. Taken together, these results suggest that activity in these regions is not contributing to the signal observed for the sources within the amygdala. Lines represent the mean±SEM. (* = *p*<0.05).

Unlike the amygdala ROI, these regions do not show bursts of gamma activity in response to unfiltered faces at ∼170 ms (Figure 6a–c). Next we exported the timecourse of activity from these ROIs and analyzed these data using the analysis strategy described above. As with the gamma oscillations, none of these regions showed Unfiltered>Filtered evoked responses at ∼170 ms (Figure 6d–f). These results suggest that we are able to localize response patterns to sources within the amygdala. Furthermore, the activity patterns observed within the amygdala are consistent with theoretical predictions [48].

### 3.5 Data Availability Statement

The summary data underlying these findings have been included as a supplemental file, accompanying this manuscript (See Data S1).

## Discussion

### 4.1 Trace Conditioning without Awareness is Possible

We trained subjects to fear unperceived faces. Consistent with previous studies, we found that subjects show learning without awareness during delay conditioning [30], [46], [54]. Furthermore, because we used faces as CSs, we were able to extend these findings to trace conditioning in which awareness is normally required. We show face-specific activation in a network of brain structures including V1, the occipital face area, and the amygdala. The amygdala exhibited large bi-phasic evoked responses and induced gamma oscillations, evoked by masked faces. These results suggest 1) that the amygdala contributes to the maintenance of visual face information, and 2) can potentially support trace conditioning, even when perception is masked.

Fear conditioning depends on plasticity in the amygdala, suggesting that components of the association between CS and UCS are formed in this structure during learning [1], [55], [56]. Interestingly, this is true even for trace conditioning [56], where the CS and UCS never actually co-occur. Therefore, there must be some alternate signal, capable of representing the CS during the trace interval. Although the hippocampus is necessary for trace conditioning [57], hippocampal neurons do not fire persistently during the trace interval [58]. In contrast, neurons in the prefrontal cortex, which is also necessary for trace conditioning [59] do show persistent firing during the trace interval [60]. Given that trace conditioning generally requires awareness, neural activity mediating this explicit CS representation may provide input for amygdala circuits. Accordingly, prefrontal regions that correlate with awareness during conditioning [61] are activated during the trace interval [3]. Interestingly, our behavioral results suggest that faces can be represented across time without awareness. Additionally, our neural data suggest that this representation could actually be mediated by the amygdala.

The idea that the amygdala can maintain visual facial information is consistent with early cellular models of learning that include a memory “trace” that reverberates throughout a cellular assembly, leading to changes in connections among the assembly neurons [62]. Furthermore, faces evoked biphasic amygdala responses during the trace interval, consistent with some formal learning models where stimuli evoke both rapid and sustained responses in processing nodes [63].

Although some studies have looked at fear learning with MEG, ours is the first to investigate conditioning without awareness. Several have shown learning related changes in sensory processing [64]–[67]. Although we observe face-specific responses in occipital regions, we do not see learning related activity, which suggests that enhanced sensory processing may be a function of explicit learning. Alternatively, the brief duration of our stimuli may have impacted our ability to show this enhancement.

One drawback to our study is that we did not directly observe CS+>CS− differences the amygdala. However, it could be that the differential amygdala response may not have been strong enough to be observed above the noise. Given that the localization of sources within deep structures with MEG is an emerging field this possibility is difficult to rule out. Alternatively, differential amygdala responses tend to occur early during training [68], and the large number of trials needed for the MEG recordings could have led to habituation [69]. Finally, faces evoke amygdala responses by default. It could be that the inhibition of these responses during unreinforced trials requires top-down inhibition, which could depend upon awareness [70].

### 4.2 Amygdala Activity during the Trace Interval may Support Learning

We showed that unfiltered faces evoke larger amygdala responses than filtered faces at ∼170 ms. Because MEG signal-to-noise is not favorable to deeper sources, very few MEG studies have successfully investigated amygdala function. Of these studies, only one used a fear conditioning paradigm. Moses and colleagues measured MEG during differential fear conditioning [66], and found larger amygdala responses for the CS+ at ∼270 ms. Interestingly, they also observed amygdala activity during the UCS period on CS+ alone trials, suggesting that the amygdala was sensitive to the onset of the CS+ and to the expectation of the UCS. Similarly, in our unfiltered group amygdala responses were bi-phasic, suggesting that amygdala activity may have a similar role here. The remaining studies either compared emotional faces to neutral faces, or faces to objects. They show that amygdala responses to emotional faces are rapid, occurring as early as 80–100 ms [71]–[73]. They are automatic, resistant to manipulations of attentional load [74], resistant to manipulations of spatial attention [71], and resistant to backward masking [73], [75]. Additionally, one study suggests that the amygdala can process emotions in low-pass filtered faces [72]. In contrast to these studies, we used source imaging to model MEG signals. We created an individual 3-D surface model of the amygdala, which complemented the standard cortical source model found in most MEG source imaging studies. To our knowledge, only one other study has used this approach [76].

Our results suggest that even neutral faces receive preferential processing from the amygdala. In contrast, some have suggested that the amygdala responds to emotional facial expressions [77]. These differences can be reconciled by a two-stage theory of amygdala function. According to this theory, the amygdala evaluates salient visual information for evidence of threat in the environment. Then, if the visual input contains specific information that has previously predicted an aversive outcome, it generates an autonomic fear reaction to prepare the organism to react to the threat.

In addition to showing face-specific evoked amygdala responses, we also show face-specific induced amygdala gamma oscillations. Interestingly, coherent gamma oscillations have been hypothesized to support object representations [49], [78], and visual awareness [79]. Our increase in gamma power is strong evidence that the amygdala plays a functional role in the maintenance of visual facial information during the trace interval. Gamma oscillations have been observed using depth electrodes implanted in the amygdala of epilepsy patients and non-human animals [51], [80], [81]. In at least two studies, gamma coherence between the amygdala and either the striatum [81] or the rhinal cortex [80] has been shown to play a role in associative learning.

### 4.3 Masked Faces Evoke Activity in Early but not Late Visual Processing Areas

Here we demonstrate face-specific responses in V1, the lingual gyrus, and the occipital face area as early as 100 ms, which replicates several previous studies. For instance, Yang and colleagues presented faces and objects while measuring electroencephalography (EEG) [82], and found synchronization between fronto-central and occipito-temporal electrodes at around 120 ms. Others have shown that upright faces evoke larger occipital P1 and M100 responses than inverted faces [83]–[85], which are not interrupted by visual masking [83]. In addition, fearful faces [86] and eye whites [87] potentiate occipital P1 responses, independent of attention [71], [73], [88].

Several previous studies EEG/MEG have demonstrated face-specific activation at ∼170 ms, which is often localized to the fusiform gyrus [82], [85], [89]. We did not observe differences in fusiform gyrus activity during the M170. There are two possible explanations for this null result. First, our experimental manipulation (high-pass filtering) was not designed to impact fusiform activity [90]. Second, our masking manipulation could have interfered with fusiform activity [83], [91]. However it is interesting to note that our amygdala responses also occurred at ∼170 ms, suggesting that these responses potentially emerge from common neural antecedents.

### 4.4 Conclusions

Under most conditions, awareness is required for trace fear conditioning. The functional role of awareness in this type of learning may be to maintain an internal representation of the CS across time so that it can become associated with the UCS. However, when a face is used as the signal for shock subjects can learn this association even if they are unable to explicitly state the predictive relationship between the CS and the UCS. This may be because the amygdala is capable of storing a representation of this stimulus across a brief trace interval. Consistent with this hypothesis, unperceived faces evoke robust amygdala responses and bursts of gamma activity during the trace interval.

## Supporting Information

Data S1 **Supplemental data summarizing behavioral data from the training and testing phases, and the recording data from the amygdala during the training phase.**
(XLSX)

Video S1 **Video showing greater responses to unfiltered than to filtered faces during the trace interval.** Colors represent the magnitude of the Unfiltered>Filtered t-test at the corresponding dipole. Warm colors represent larger responses to unfiltered faces than to filtered faces. Cool colors represent larger responses to filtered faces than to unfiltered faces.
(WMV)
